# Successful treatment of subdural hemorrhage and retinal hemorrhage in childhood-onset systemic lupus erythematosus associated with thrombocytopenia: Case report

**DOI:** 10.1097/MD.0000000000024231

**Published:** 2021-01-15

**Authors:** Yu Wen, Ping Lu, Huiling Lu, Xiufen Hu

**Affiliations:** Department of Pediatrics, Tongji Hospital Affiliated to Tongji Medical College, Huazhong University of Science and Technology, Wuhan, China.

**Keywords:** childhood-onset systemic lupus erythematosus, retinal hemorrhage, subdural hemorrhage, thrombocytopenia

## Abstract

**Introduction:**

Thrombocytopenia (TP) is a common complication of childhood-onset systemic lupus erythematosus (SLE), and can range from mild to life-threatening. However, severe TP with multiple hemorrhagic complications is very rare and often predicts a poor prognosis. We describe a 12-year-old Chinese girl who had a history of idiopathic thrombocytopenic purpura who developed SLE that presented as subdural hemorrhage and retinal hemorrhage because of severe TP.

**Patient concerns:**

A 12-year-old girl was admitted into our hospital because of fever, purpura, and gum bleeding lasting for 12 days. She had a history of idiopathic thrombocytopenic purpura 2 years ago previously.

**Diagnosis:**

SLE was diagnosed according to American College of Rheumatology classification criteria. Subdural hemorrhage and retinal hemorrhage were diagnosed based on brain MRI and funduscopy. Severe TP was defined as platelet count <20 × 10^9^/L.

**Interventions:**

She was treated first with intravenous immunoglobulin, but it was not efficacious. High-dose methylprednisolone showed short-term efficacy. Then, she was given a glucocorticoid and cyclosporine A plus mycophenolate mofetil.

**Outcomes:**

Fever, purpura, and gum bleeding were resolved before hospital discharge. Subdural hemorrhage and left hemorrhagic retinopathy were improved remarkably. She had a durable response to refractory TP with no adverse effects during >1-year follow-up.

**Conclusion:**

Isolated TP may be an early symptom of childhood-onset SLE . A child with severe TP is prone to develop life-threatening hemorrhagic complications. Glucocorticoids and combined immunosuppressive drugs had a durable response to refractory TP in this patient with no adverse effects.

## 1 Introduction

Systemic lupus erythematosus (SLE) is a multisystem illness. Patients with childhood-onset systemic lupus erythematosus (c-SLE) have a more severe course and organ damage than patients with adult-onset SLE.^[1,2]^ Up to 40% of patients with SLE develop thrombocytopenia (TP). TP is a reliable marker suggesting active disease and often predicts a poor prognosis.^[3]^ Severe TP is relatively uncommon, but it can result in visceral hemorrhage and death.

Although spared from kidney involvement, our pediatric patient developed intracranial and retinal hemorrhages, which were vision-threatening. To date, such a pediatric case has not been reported before.

## 2 Case report

A 12-year-old girl with a history of idiopathic thrombocytopenic purpura of 2-year duration received intravenous immunoglobulin (IVIG; 1 g/kg bodyweight) treatment for prolonged fever, purpura, gum bleeding, and TP at a local hospital, but showed no response. She developed myalgia, arthralgia, headache, and severe TP (platelet count, 2 × 10^9^/L) lasting for 12 days and was transferred to our hospital.

Upon physical examination, dental ulcers, gum bleeding, purpura on lower extremities, mild hepatomegaly, arthritis on elbow joints, and painless loss of vision in her left eye were noted. Neurological findings appeared normal.

Complete blood cell counts showed a normal white blood cell count (8.28 × 10^9^/L), normocytic normochromic anemia (hemoglobin, 84 g/L) and severe TP (platelet count, 6 × 10^9^/L). Biochemical investigations showed levels of alanine aminotransferase to be 43 U/L, aspartate aminotransferase to be 133 U/L, lactate dehydrogenase to be 800 U/L, and with normal blood urea nitrogen and serum creatinine. Other laboratory findings revealed increased erythrocyte sedimentation rate (117 mm/h), hypocomplementemia (complement C3, 0.4 g/L; complement C4, 0.08 g/L), positivity for serum anti-nuclear antibodies (1:3200), anti-Sm, anti-SS-A, anti-Sm/nRNP, anti-chromatin, anti-RNP 68, anti-RNP A antibodies, and negative for serum anti-dsDNA antibody. The Coombs’ test was 2+, and the patient was negative for antiphospholipid antibody, lupus anticoagulant, and antiplatelet antibody. The activated partial thromboplastin time was 36.6 second, and prothrombin time was 14.2 second.

**Figure 1:**
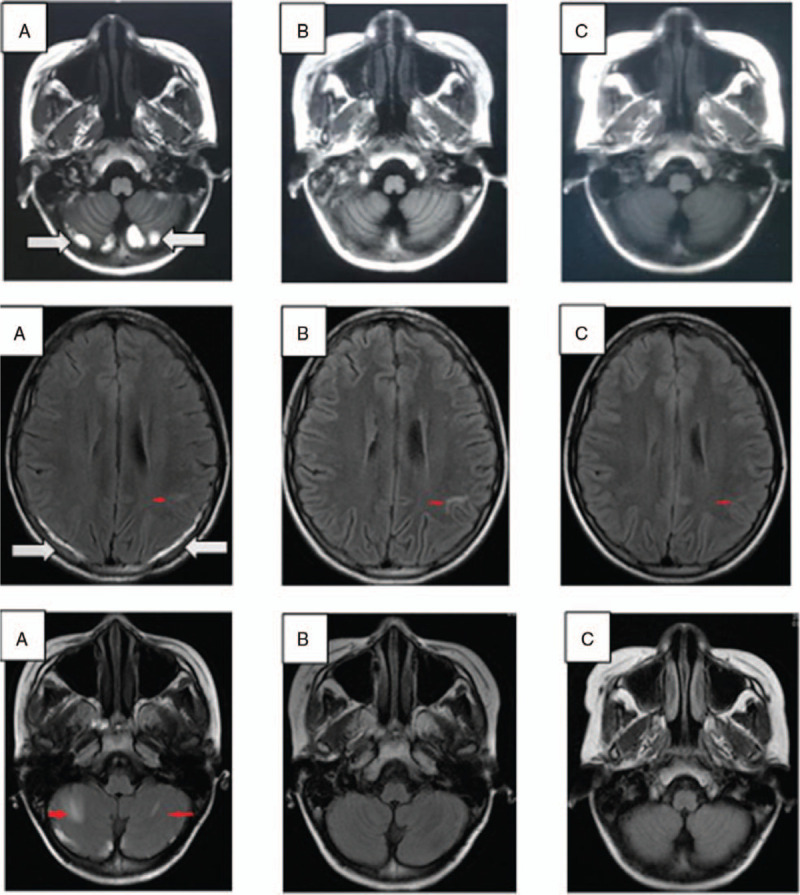
Magnetic resonance imaging (MRI) of the brain at different times. (A) Upon presentation, MRI showed a subacute subdural hemorrhage (white arrow) and multiple demyelinating lesions (red arrow) in bilateral cerebral hemispheres and cerebellum. (B) One month later and (C) 4 months later, MRI showed that the subdural hemorrhage had been absorbed completely, and multiple demyelinating lesions became smaller or disappeared in the cerebellum.

**Figure 2:**
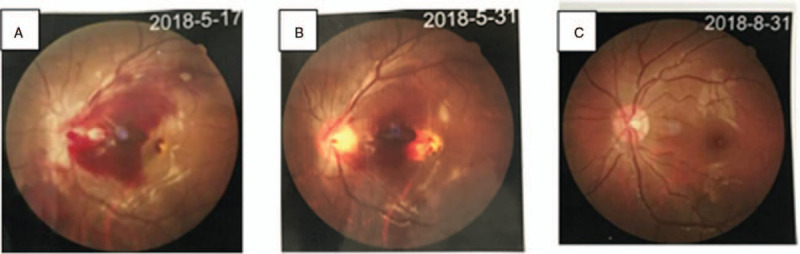
Fundus images. (A) Massive retinal hemorrhage on her left eye upon presentation. (B) The retinal hemorrhage on her left eye was absorbed partially after 2 weeks. (C) The retinal hemorrhage on her left eye was absorbed completely after 3 months.

Urinalyses were normal. Ultrasound of the abdomen revealed hepatosplenomegaly. Subsequent magnetic resonance imaging of the head showed a subacute subdural hemorrhage in bilateral cerebral hemispheres and cerebellum, and multiple demyelinating lesions in the bilateral frontotemporal cortex and bilateral cerebellar hemispheres (Fig. 1A). Funduscopic examination indicated severe retinal hemorrhage in the left eye (Fig. 2A). Based on these data, a diagnosis of severe SLE was established.

**Figure 3:**
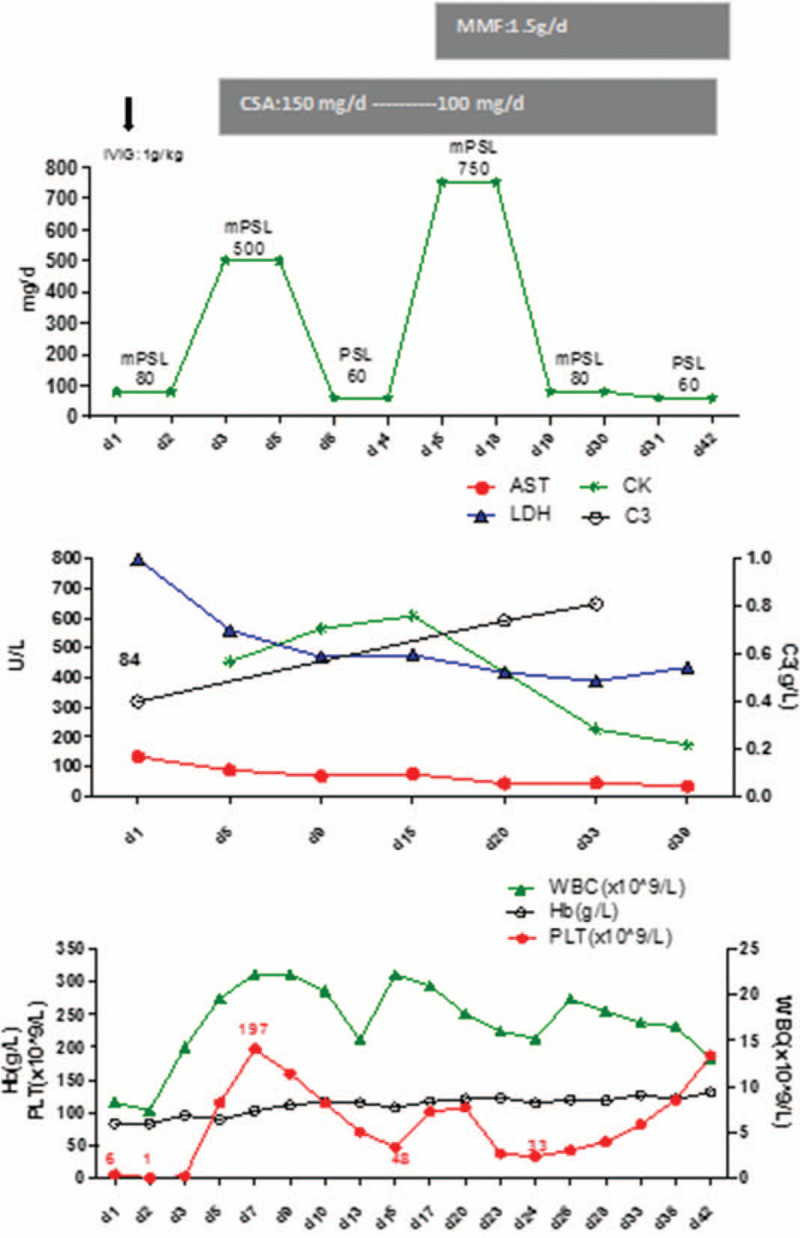
The clinical course of our patient in hospital.

Emergency treatment was initiated with platelet transfusion, IVIG (1 g/kg) and 2 pulses of methylprednisolone (mPSL) with 80 mg/pulse intravenous infusion. Nonetheless, the platelet number failed to increase. On day-3 of hospitalization, she was given high-dose (500 mg) mPSL daily for 3 days followed by high-dose (60 mg/d) prednisolone. The platelet count continued to decrease, so she was also given cyclosporine-A (CSA) (75 mg, q12 hour, p.o.). After intravenous high-dose mPSL, the fever resolved, and the anemia and TP improved. On day-7 of hospitalization, the hemoglobin level increased to 104 g/L, platelet count returned to 197 × 10^9^/L but decreased to 48 × 10^9^/L on day-15. Due to high SLE activity (Systemic Lupus Erythematosus Disease Activity Index was 30) and the very few megakaryocytes in bone marrow (1 megakaryocyte count per slide), she was treated with high-dose (750 mg/d for 4 days) mPSL plus mycophenolate mofetil (MMF). The platelet count continued to fluctuate significantly. Fortunately, her symptoms were much improved. By day-26 of hospitalization, the platelet count began to increase steadily and reached 187 × 10^9^/L on day-42. Then, she was discharged and continued with, CSA, and MMF. Her clinical course in hospital is illustrated in Figure 3.

The subdural hemorrhage (Fig. 1B, C) and left hemorrhagic retinopathy (Fig. 2B and 2C) improved remarkably. Her platelet count decreased to 47 × 10^9^/L because of a respiratory infection 6 months after discharge from hospital, but recovered to 148 × 10^9^/L after 1-week antibiotic treatment. The patient was followed up for >1 year after hospital discharge. The dose of oral drugs was tapered gradually. Relevant side effects did not appear.

## 3 Discussion

TP is a common manifestation in SLE, and often predicts a poor prognosis. The pathogenesis of TP in SLE is not known. The presumed mechanisms include: platelet destruction and deletion by autoantibodies in the peripheral circulation; inhibition of the development and maturation of megakaryocytes in bone marrow; consumption of platelets because of thrombotic microangiopathy/thrombotic thrombocytopenic purpura (TTP); secondary antiphospholipid syndrome (APS). These mechanisms often coexist.^[3]^ Inhibition of the development and maturation of megakaryocytes in bone marrow is an important factor. Zhao et al^[3]^ reported that patients with a megakaryocyte count in bone marrow <20/slide have a poor clinical response to immunotherapy. In our case, the megakaryocyte number decreased significantly, with 1 megakaryocyte count per slide justifying that TP was refractory to immunotherapy.

Retinal hemorrhage and subdural hemorrhage occurring together are unusual in SLE (especially in c-SLE). About one-third of SLE patients develop ocular manifestations, with 7% to 29% showing retinal involvement. Retinopathy is probably caused by “cotton wool” spots, retinal haemorrhage and microvascular infarcts.^[4,5]^ SLE patients are prone to develop hemorrhagic complications. Spontaneous intracranial hemorrhage is the most serious complication of TP, and is potentially life-threatening. The mechanisms of intracranial hemorrhage are incompletely understood, but several related causes have been summarized,^[6]^ including hypercholesterolemia, hypertension, prolonged treatment with corticosteroids, angiopathy, and aneurysms caused by SLE. Moreover, TP is also an important risk factor. In a single-center retrospective study, 26 out of 6653 SLE cases had intracranial hemorrhage. In these 26 cases, 53.8% had TP, and TP was an independent risk factor for intracranial hemorrhage induced by SLE.^[7]^ That finding is consistent with that of our patient, and the hemorrhages were absorbed while the platelet count returned to normal.

TP treatment in SLE is dependent upon the presentation of systemic disease. Treatment of TP in SLE needs to be specific. Glucocorticoids are regarded as first-line therapy in severe TP but very few patients have sustained remission. IVIG shows rapid, but not long-term, efficacy in patients suffering active bleeding. Hence, immunosuppressive therapy may be warranted.^[8]^ One case of bilateral hemorrhagic retinopathy treated with azathioprine showed the disappearance of Roth spots within 6 weeks of treatment.^[9]^ CSA treatment in patients presenting with TP associated with SLE has shown optimal relief.^[10]^ Broad-spectrum immunosuppressants such as cyclophosphamide have been found to be efficacious in patients with refractory TP in SLE.^[11]^ Some case studies have shown that MMF and tacrolimus can be used to treat TP successfully.^[11,12,13]^ However, some TP patients are refractory to conventional therapies. Biologics could be a good choice for patients with TP in SLE. In a study by Olfat and colleagues,^[14]^ 24 refractory patients with c-SLE were treated with rituximab, of whom 16 had TP, 5 had hemolytic anemia, and 3 had both. Only 1 patient failed to respond to rituximab. All patients tolerated rituximab treatment. However, caution should be exercised when considering rituximab for patients with preexisting hypogammaglobulinemia. According to Lei and colleagues, c-SLE patients intolerant of rituximab who used another B cell-depleting agent, ofatumumab, also obtained a therapeutic effect.^[15]^ Additional treatments, such as plasmapheresis and thrombopoietin receptor agonists, can be efficacious in patients with refractory TP and active bleeding. For some patients with refractory TP non-responsive to medical therapies, splenectomy may be an important alternative.^[16]^ Successful treatment for a-SLE is well documented, but not for c-SLE. Our case had severe TP with potential organ bleeding, so we initiated emergency treatment at an early stage: platelet transfusion, IVIG, mPSL, and CSA. The platelet count fluctuated significantly, so we added MMF which, ultimately, improved TP.

## 4 Conclusions

Spontaneous subdural hemorrhage with retinal hemorrhage as a consequence of TP in c-SLE is rare. However, we successfully treated our patient with a glucocorticoid combined with 2 immunosuppressants. We consider this regimen to be safe and efficacious treatment in severe TP of c-SLE.

## Author contributions

**Conceptualization:** Ping Lu, Huiling Lu.

**Data curation:** Ping Lu, Huiling Lu.

**Project administration:** Yu Wen, Ping Lu, Huiling Lu.

**Supervision:** Xiufen Hu.

**Writing – original draft:** Yu Wen.

**Writing – review & editing:** Yu Wen, Xiufen Hu.

## Abbreviations

Abbreviations: CSA = cyclosporin-A, c-SLE = childhood-onset systemic lupus erythematous, IVIG = intravenous immunoglobulin, MMF = mycophenolate mofetil, mPSL = methylprednisolone, SLE = systemic lupus erythematosus, TP = thrombocytopenia.
